# Increased Risk of Hospitalization Related to Motor Vehicle Accidents Among People Taking Zolpidem: A Case–Crossover Study

**DOI:** 10.2188/jea.JE20090195

**Published:** 2011-01-05

**Authors:** Yao-Hsu Yang, Jung-Nien Lai, Chang-Hsing Lee, Jung-Der Wang, Pau-Chung Chen

**Affiliations:** 1 Institute of Occupational Medicine and Industrial Hygiene, National Taiwan University College of Public Health, Taipei, Taiwan; 2 Department of Traditional Chinese Medicine, Chang Gung Memorial Hospital, Chia-Yi, Taiwan; 3 Institute of Traditional Medicine, School of Medicine, National Yang-Ming University, Taipei, Taiwan; 4 Department of Obstetrics and Gynecology and Department of Chinese Medicine, Taipei City Hospital, Yangming Branch, Taipei, Taiwan; 5 Department of Occupational Medicine, Ton Yen General Hospital, Hsin-Chu County, Taiwan; 6 Department of Internal Medicine, National Taiwan University Hospital, Taipei, Taiwan; 7 Department of Environmental and Occupational Medicine, National Taiwan University Hospital, Taipei, Taiwan

**Keywords:** zolpidem, benzodiazepines, motor vehicle accidents, case–crossover design, pharmacoepidemiology

## Abstract

**Background:**

Several epidemiological and experimental studies have found a positive association between the risk of motor vehicle accidents (MVAs) and use of zopiclone and benzodiazepines. There is, however, little evidence of any risk of MVA attributable to the use of zolpidem 1 day before such accidents. We attempted to determine whether the use of zolpidem 1 day before is associated with an increased risk of an MVA.

**Methods:**

Using a 1-million-person randomly sampled cohort from the Taiwan National Health Insurance reimbursement database, 12 929 subjects were identified as having been hospitalized between 1998 and 2004 due to an MVA. Using a case–crossover design, we selected the day before an MVA as the case period for each subject, and the 91st, 182nd, and 273rd days before the case period as 3 retrospective control periods. Conditional logistical regression models were constructed to calculate the odds ratio (OR) of having an MVA and the exposure of zolpidem 1 day before. We calculated doses of benzodiazepines, zopiclone, and zolpidem based on their defined daily dose.

**Results:**

The adjusted OR for involvement in an MVA after taking 1 defined daily dose of zolpidem was 1.74 (95% confidence interval: 1.25–2.43). There were also positive effects for different washout periods and cumulative doses at 7, 14, 21, and 28 days before the occurrence of an MVA.

**Conclusions:**

Use of zolpidem 1 day before might be associated with an increased risk of MVA. Thus, precautionary warnings should be provided when prescribing zolpidem.

## INTRODUCTION

Insomnia is a frequent complaint in modern society, and zolpidem, zopiclone, and benzodiazepines (BZDs) are among the most common medications for treating this condition.^1^ The pharmacological mechanisms of non-benzodiazepines (non-BZDs) such as zolpidem and zopiclone are similar to those of benzodiazepines; however, non-BZDs are far more selective in the receptor that they target and have fewer side effects than BZDs. It is generally accepted that, as compared with BZDs, the use of non-BZDs to treat insomnia results in fewer residual effects on the day after treatment.^2^ Several recent epidemiological studies have noted associations between the use of zopiclone and BZDs and an increased risk of motor vehicle accidents,^3^^–^^8^ but there is very little evidence regarding the risk of motor vehicle accidents after zolpidem use.^3^

Some experimental studies observed impaired driving ability and psychomotor functions shortly after zolpidem use^9^^,^^10^; however, other similar studies found no association with functional impairment when zolpidem was used more than 10 to 11 hours before assessment.^11^^–^^13^ Thus, it has generally been assumed that zolpidem taken at bedtime will not affect driving safety on the following day. The primary aim of this study was to determine whether the use of zolpidem on the day before a motor vehicle accident was associated with an increased risk of such accidents.

## METHODS

### Data source

The sampling cohort dataset was obtained from the Taiwanese National Health Insurance (NHI) research database. The reimbursement data files of the NHI, which are compiled and managed by the National Health Research Institutes, are comprehensive and contain information on all medications prescribed to all residents of Taiwan.

The dataset used in this study was compiled from a random sampling of 1 million individuals from the total population of 23 400 826 people enrolled in the NHI. The reimbursement data collected for analysis in this study cover the period from 1 January 1997 to 31 December 2004. We utilized databases for admissions and outpatient visits of the sample cohort, both of which include information on patient characteristics, including sex, date of birth, date of admission, date of discharge, dates of visits, admission diagnosis, and outpatient visit diagnosis. The data files also contain information on patient prescriptions, including the names of prescribed drugs, dosage, duration of prescription, and total expenditure.

This study adhered to strict confidentiality guidelines, in accordance with regulations regarding personal electronic data protection, and was approved by the ethics review board of the National Taiwan University College of Public Health.

### Study subjects

Using the diagnostic variable in admissions data from the NHI database, we selected motor vehicle accidents resulting in hospitalization, ie, codes E810 to E825 of the International Classification of Disease, 9th Revision, Clinical Modification (ICD-9-CM), after excluding all persons involved in accidents coded with fifth-digit subdivisions of .1, .3, .4, .5, and .7, which represent accidents involving non-drivers. Because of our concern that a history of a motor vehicle accident might change a subject’s driving behavior, we included only first accidents in the analysis. The admissions database also indicated whether the admission was attributable to a traffic accident.

In addition to the 2 abovementioned inclusion criteria, subjects had to be 18 years of age or older, with an initial hospital admission attributable to a motor vehicle accident that occurred between 1998 and 2004. Medication records of individuals could be traced back 1 year before hospitalization, using the NHI database for 1997 to 2004. From our initial random sample cohort of 1 million people extracted from the NHI database, we ultimately identified a total of 12 929 subjects who had been involved in motor vehicle accidents resulting in hospitalization during the period under examination.

### Case–crossover design

We used a case–crossover design, with each case acting as its own control, to take into consideration potential confounding factors attributable to comorbid conditions, individual lifestyle, and driving habits.^14^ Such a design is usually applicable to the study of adverse drug reactions involving a transient exposure followed by a short, but steep, increase in risk.

### Medication use

All data on medications used in this study sample were retrieved from the NHI database. In accordance with the Anatomical Therapeutic Chemical (ATC) classification of drugs, we selected zolpidem, zopiclone, BZD anxiolytics, and BZD hypnotics as the major risk factors of interest, with oxazolam included in the category of BZD anxiolytics. The BZDs were then classified into 2 groups based upon their half-life. The first group comprised those drugs with a half-life longer than 24 hours, which was defined as the long-half-life group. These included chlordiazepoxide, cloxazolam, diazepam, flurazepam, medazepam, nordazepam, potassium clorazepate, and oxazolam. The second group comprised BZDs with a half-life shorter than 24 hours, which was defined as the short-half-life group. These included alprazolam, bromazepam, brotizolam, clobazam, estazolam, fludiazepam, flunitrazepam, lorazepam, lormetazepam, midazolam, nitrazepam, oxazepam, temazepam, and triazolam.

A common problem when evaluating drugs with different strengths and potencies is that it is uninformative to compare the same weights of different drugs. The defined daily dose (DDD) is a unit for measuring a prescribed amount of drug: it is the assumed average maintenance dose per day of a drug consumed for its main indication in adults. We used the following formula to standardize drug use in the present study: (total amount of drug)/(amount of drug in a DDD) = number of DDDs. By using the DDD to standardize different drugs, we were able to compare drugs based upon the same standard.

Given that variations in both the dose and potency of each of the different prescribed medications may have diverse effects on driving ability and psychomotor performance in different individuals, all dosages for each subject were counted using data on their personal medication and then standardized at the DDD rate. Thus, we were able to use the same unit to compare the risk of motor vehicle accidents for different drugs.

Any use of other medications that could increase the risk of motor vehicle accidents was treated as a covariate; these included other sedatives/hypnotics, anticonvulsants, antidepressants, other psychoactive drugs, centrally acting muscle relaxants, opioid analgesics, and antihistamines.

### Cases, controls, and washout periods

For each subject in this study, the case–crossover design matched 1 case period to 3 earlier control periods. We selected the day before the motor vehicle accident as the case period and the 91st, 182nd, and 273rd days before the case period as the 3 control periods for each subject. This ensured that the day of the week was the same for the case and control periods for each subject.

A washout period was established between the case period and each of the 3 control periods to reduce the likelihood of overlapping prescriptions between these periods. Because the mean duration (± standard deviation) of prescriptions for zopiclone, zolpidem, and BZDs was 11.7 ± 10.4 days, and as no medication was prescribed to any of the patients in our study for longer than 13 weeks, we selected 13 weeks as the washout period.

### Statistical analysis

Our modeling procedure involved the use of 1-to-3 matched conditional logistic regressions to calculate the odds ratio (OR) of involvement in motor vehicle accidents resulting in hospitalization in those cases where there was use of zolpidem, zopiclone, or BZDs on the day before the accident. Adjusted ORs and 95% confidence intervals (95% CIs) were calculated using DDD units of zolpidem, zopiclone, long-half-life BZDs, and short-half-life BZDs, which were adjusted for use of other types of medication that might be potentially related to motor vehicle accidents and for related risk factors. Therefore, we were able to determine how much an increase of 1 DDD augmented the risk of motor vehicle accidents. Analysis of the data in the study was carried out using SAS software, version 9.1 (SAS Institute Inc. Cary, NC, USA).

## RESULTS

From our dataset of 1 million individuals randomly selected from the NHI database between the years of 1998 and 2004, we identified a total of 12 929 subjects who had been involved in motor vehicle accidents and satisfied the criteria specified in the Methods section. The characteristics of the study subjects are summarized in Table 1.

**Table 1.** Characteristics of study subjects

Characteristic	No.	%
Sex		
Male	7971	61.7
Female	4958	38.3

Age, years		
18–45	7601	58.8
46–64	3698	28.6
≥65	1630	12.6

Day of motor vehicle accident		
Sunday	1544	12.0
Monday	2033	15.7
Tuesday	1903	14.7
Wednesday	1860	14.4
Thursday	1843	14.3
Friday	1973	15.3
Saturday	1773	13.7

No. of prescription days during the 1 year before the motor vehicle accident		
0	9449	73.1
1–28	2208	17.1
29–56	356	2.8
57–84	172	1.3
≥85	744	5.8

Total	12 929	100.0

Males accounted for approximately 62% of our total sample. Furthermore, the proportion of motor vehicle accidents by day of the week was slightly lower on weekends (particularly on Sundays) as compared with weekdays. More than a quarter of the subjects had taken zopiclone, zolpidem, or a BZD at least once during the 1-year period before their motor vehicle accidents.

Table 2 shows the adjusted ORs for involvement in a motor vehicle accident, by DDD of zolpidem, zopiclone, and BZDs on the day before hospitalization. An increased OR was observed for zolpidem after adjustment for other types of medications and related risk factors. The ORs for involvement in a motor vehicle accident after use of a non-BZD on the day before the accident were 1.74 for zolpidem (95% CI: 1.25–2.43) and 1.55 for zopiclone (0.98–2.45). Similarly, the adjusted ORs for involvement in a motor vehicle accident after use of BZDs on the day before the accident were 1.74 per DDD for long-half-life BZDs (1.26–2.40) and 1.13 per DDD for short-half-life BZDs (1.04–1.23).

**Table 2.** Adjusted odds ratios (ORs) and 95% confidence intervals (CIs) of motor vehicle accidents per defined daily dose of benzodiazepines, zopiclone, and zolpidem taken 1 day before hospitalization, stratified by age and sex

Medication	All subjects	Sex		Age, years		
	
		Male	Female	18–45	46–64	≥65
Zolpidem						
No. exposed–case period^a^	87	49	38	30	38	19
No. exposed–control period^b^	170	90	80	62	68	40
Adjusted OR^c^	1.74	2.08	1.40	1.56	2.06	1.70
95% CI	1.25–2.43	1.30–3.32	0.86–2.28	0.88–2.75	1.19–3.55	0.88–3.29

Zopiclone						
No. exposed–case period^a^	36	18	18	13	13	10
No. exposed–control period^b^	71	40	31	29	26	16
Adjusted OR^c^	1.55	1.46	1.68	1.38	1.53	3.23
95% CI	0.98–2.45	0.75–2.84	0.88–3.19	0.65–2.94	0.78–3.00	0.95–10.97

Long-half-life benzodiazepine^d^						
No. exposed–case period^a^	149	97	52	45	56	48
No. exposed–control period^b^	331	203	128	84	150	97
Adjusted OR^c^	1.74	1.96	1.37	1.71	0.94	4.41
95% CI	1.26–2.40	1.30–2.95	0.80–2.35	1.04–2.82	0.53–1.68	2.16–8.98

Short-half-life benzodiazepine^e^						
No. exposed–case period^a^	558	324	234	171	241	146
No. exposed–control period^b^	1321	724	597	396	561	364
Adjusted OR^c^	1.13	1.16	1.09	1.22	1.20	0.99
95% CI	1.04–1.23	1.04–1.30	0.95–1.24	1.04–1.42	1.05–1.37	0.84–1.16

^a^No. of individuals who had a motor vehicle accident while exposed.

^b^No. of individuals exposed during the 91st, 182nd, and 273rd days before the case period.

^c^Odds ratios are adjusted for the other 3 of the 4 drugs in the table and for other drugs that might be associated with motor vehicle accidents, including antihistamines, anticonvulsants, antidepressants, other sedatives/hypnotics, other psychoactive drugs, centrally acting muscle relaxants, and opioid analgesics.

^d^Half-life ≥24 hours.

^e^Half-life <24 hours.

The highest OR was for use of long-half-life BZDs in subjects aged 65 years or older. Among the 4 different medication types, short-half-life BZDs appeared to have a lesser effect than the other medication types in all age strata except individuals aged 46 to 64 years. Males who took zolpidem or BZDs had higher ORs for motor vehicle accidents than did females who took these medications.

We also examined the effects of cumulative doses of zolpidem, zopiclone, and BZDs on adjusted ORs for motor vehicle accident, with cumulative doses individually summed for periods of 7, 14, 21, and 28 days before the occurrence of the motor vehicle accident and for the 3 control periods; the results are summarized in Table 3. Within the 4 different cumulative periods described above, as compared with the lower OR for the 7-day accumulation of the DDD for short-half-life BZDs, the OR for zolpidem, zopiclone, and long-half-life BZDs was 1.08. However, as the number of accumulated days increased, the ORs decreased for each DDD of zolpidem, zopiclone, and long-half-life BZDs; indeed, for the 28-day period, there were no differences in the ORs for the 4 medication types. Thus, there was a general trend toward a reduced risk for each DDD, and the magnitude of this decline appeared to be slightly greater for zolpidem and zopiclone, as compared with short-half-life BZDs.

**Table 3.** Adjusted odds ratios (ORs) and 95% confidence intervals (CIs) of motor vehicle accident per defined daily dose of benzodiazepines, zopiclone, and zolpidem, stratified by duration of administration before hospitalization

Medication	Duration of administration			

	7 days	14 days	21 days	28 days
Zolpidem				
No. exposed–case period^a^	96	111	118	125
No. exposed–control period^b^	199	222	247	267
Adjusted OR^c^	1.08	1.05	1.03	1.03
95% CI	1.03–1.13	1.02–1.07	1.01–1.05	1.01–1.04

Zopiclone				
No. exposed–case period^a^	41	42	47	52
No. exposed–control period^b^	80	96	107	111
Adjusted OR^c^	1.08	1.03	1.02	1.02
95% CI	1.00–1.16	0.99–1.08	0.99–1.05	0.99–1.04

Long-half-life benzodiazepine^d^				
No. exposed–case period^a^	228	291	334	373
No. exposed–control period^b^	520	694	820	962
Adjusted OR^c^	1.08	1.05	1.04	1.03
95% CI	1.02–1.14	1.02–1.08	1.01–1.06	1.01–1.05

Short-half-life benzodiazepine^e^				
No. exposed–case period^a^	676	773	835	903
No. exposed–control period^b^	1635	1928	2146	2329
Adjusted OR^c^	1.02	1.01	1.01	1.01
95% CI	1.01–1.04	1.01–1.02	1.00–1.02	1.00–1.01

^a^No. of individuals exposed for the 4 respective cumulative periods before motor vehicle accidents.

^b^No. of individuals exposed for the 4 respective cumulative periods before the 91st, 182nd, and 273rd days before a motor vehicle accident.

^c^Odds ratios are adjusted for the other 3 of the 4 drugs in the table and for other drugs that might be associated with motor vehicle accidents, including antihistamines, anticonvulsants, antidepressants, other sedatives/hypnotics, other psychoactive drugs, centrally acting muscle relaxants, and opioid analgesics.

^d^Half-life ≥24 hours.

^e^Half-life <24 hours.

Various other washout periods, ranging from 9 to 17 weeks, were also analyzed to identify any fluctuations in ORs. The adjusted ORs (with 95% CIs) are shown in the Figure. With respect to zopiclone, the adjusted OR showed a decreasing trend from a washout period of 9 weeks to one of 11 weeks; there were no other major fluctuations in relation to washout period. The other adjusted ORs also showed no major fluctuations with respect to washout period.

**Figure.**
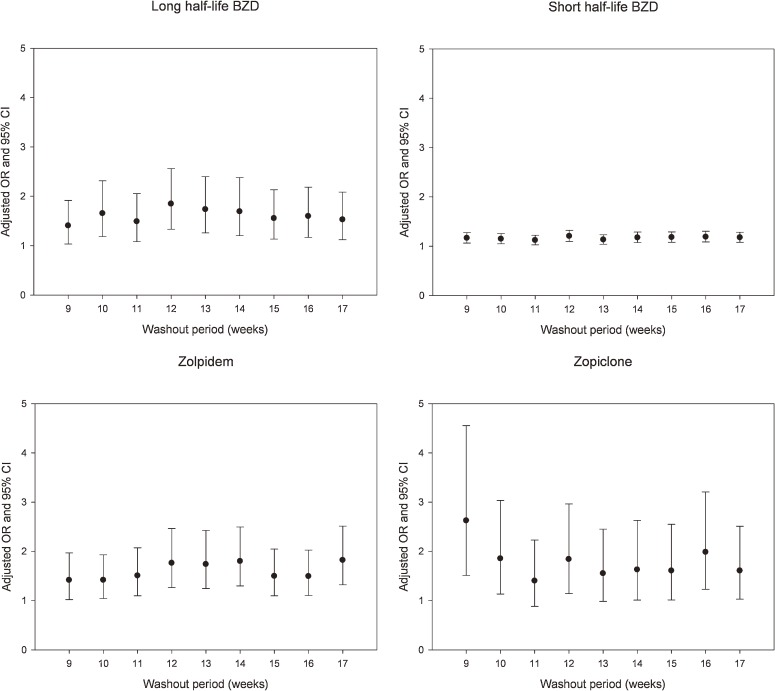
Adjusted odds ratios for hospitalization due to motor vehicle accident after use of a daily defined dose of zolpidem, zopiclone, and benzodiazepines (BZDs) on the day before the accident, by duration of washout period. Abbreviations: CI, confidence interval; OR, odds ratio.

## DISCUSSION

In the present study, we found a positive association between the risk of motor vehicle accidents and zolpidem use on the day before such accidents (Table 2). In addition, adjusted ORs for involvement in motor vehicle accidents were higher for long-half-life BZDs than for short-half-life BZDs, a finding which largely corresponds with those of earlier epidemiological studies.^5^^–^^7^ Although zopiclone was associated with an increased risk of motor vehicle accidents in several studies,^3^^,^^6^ the OR for zopiclone was not statistically significant in the present study. However, this is likely due to the small number of exposed individuals in this study.

Because the duration of washout periods ranging from 9 to 17 weeks was not associated with a significant difference in overall risk (with the exception of a washout period of 9–11 weeks for zopiclone; Figure), it is clear that the increased risk associated with zolpidem use cannot be explained by the choice of a control period with different washout periods. In addition, a sufficiently long washout period is necessary for two reasons. First, without an adequately long washout period, a case–crossover design is likely to underestimate risks in people receiving long-term therapy, because such individuals might take zolpidem throughout the case period and would thus have the same exposure in the previous control period. Secondly, within the first few weeks after initiation of drug use, individuals develop a degree of tolerance to the psychomotor-impairing effects of the drug.^15^ Without a sufficiently long washout period, tolerance is more likely to be present in the case period than in the control period. Therefore, we selected 13 weeks as a washout period that was adequate to avoid underestimation caused by long-term therapy and reduce the effects of tolerance,^4^^,^^7^ particularly in the elderly.^5^

The proportion of motor vehicle accidents by day of the week in Taiwan was slightly lower on weekends (particularly on Sundays) than on weekdays. To eliminate any potential confounding related to varying daily activities, we designed the study so that the day of the week selected for control periods was the same as that for case periods. We also adjusted for use of other types of medications that could be related to motor vehicle accidents.

Because adjustments were made for other types of medications that could be related to motor vehicle accidents in the model construction, any confounding by these factors was accounted for. Furthermore, since the case–crossover design accounts for various personal factors, such as alcohol intake, driving behavior, and comorbid conditions,^16^^–^^18^ we tentatively conclude that zolpidem use is the only explanation for the increased risk of motor vehicle accidents. In addition, we found an association between BZD use and increased risk of motor vehicle accidents, which is in line with previous epidemiological studies.^3^^–^^8^

Because pharmacokinetic and pharmacodynamic changes are related to increased age,^19^^,^^20^ it is possible that elderly adults are more susceptible to the effects of zolpidem because of slower excretion of its metabolites; however, as shown in Table 2, zolpidem was not associated with a statistically significant increase in risk for those aged 65 years or older, probably because zolpidem use was uncommon among elderly subjects in the present study. Nevertheless, we found high ORs for long-half-life BZDs among subjects older than 65 years, which suggests that it may not be safe for elderly people to drive after regular use of long-half-life BZDs.

Although several reports have found insignificant or limited effects on psychomotor performance after bedtime administration of zolpidem,^11^^,^^12^^,^^21^ the subjects involved in these experimental studies were mostly young adults, and the tests were usually conducted on driving simulators. However, the use of such simulators may not be comparable to the driving conditions of Taiwan and other less developed countries, where scooters and motorcycles are extremely common modes of transport. Therefore, we must treat previous findings on this question with considerable care and, when prescribing zolpidem to the groups represented in our study, advise patients of the potential adverse effects arising from the use of such medication.

There are several limitations of this study that must be taken into consideration. First, the study included only motor vehicle accidents resulting in hospitalization, which might have led to underrepresentation of other, less severe cases or fatal cases that had no admission record.

Secondly, because our data do not provide detailed contextual information on these motor vehicle accidents, we were unable to differentiate between cases in which subjects were struck by other drivers and those in which the subjects themselves may have been culpable. Nevertheless, those cases in which the subjects were struck by others might still have resulted from the subject’s use of hypnotics, because the medication could have affected their responses or driving behavior, thereby making them less able to avoid such accidents.

Third, although the 1997–2004 reimbursement dataset showed that more than 90% of all zolpidem prescriptions were intended for nighttime administration (or *hora somni*), there is no way to verify exactly when the study subjects actually took the medicine. However, since the major indication is treatment of insomnia, we were still able to identify an association between increased risk of motor vehicle accidents and use of zolpidem on the day before such accidents. Because some people might consume more than one drug at the same time, the possibility of drug interactions is a limitation that could not be completely adjusted for in the statistical model.

Finally, there was no information in the database on the alcohol intake or driving habits of the subjects. The case–crossover design assumes that the alcohol use and driving habits of subjects remained stable during the period of observation, ie, the 1-year period before their injury. We believe that it is highly likely that alcohol use and driving habits did not change and thus did not contribute to the association between drug use and motor vehicle accidents.

## CONCLUSIONS

Our findings suggest that, among Taiwanese, there is a positive association between the risk of motor vehicle accidents and the use of zolpidem, BZDs, and possibly zopiclone, on the day before the occurrence of such accidents. We recommend that further studies be conducted to comprehensively examine this association, and that precautionary warnings should be provided to all patients who are prescribed these medications.
